# Spontaneous tumors in dogs and cats in Algeria: a dual-source descriptive study of practitioner-reported patterns and histopathology-submitted cases

**DOI:** 10.1186/s12917-026-05856-3

**Published:** 2026-08-24

**Authors:** Aya Sofia Bouchoukh, Said Boukhechem, Hithem Bougherara, Aimene Zakaria Chaabi

**Affiliations:** Gestion de la Santé et Productions Animales Research Laboratory, Institut des Sciences Vétérinaires El-Khroub, Université de Constantine 1 Frères Mentouri, Constantine, Algeria

**Keywords:** Canine tumors, Feline tumors, Companion animals, Veterinary oncology, Histopathology, Mammary tumors, Veterinary cancer surveillance, Diagnostic availability, Algeria

## Abstract

**Background:**

Algeria has no documented national companion-animal cancer registry, and limited information is available on tumors encountered in veterinary practice or reaching histopathological confirmation. This dual-source descriptive study combined two independently analyzed components: a histopathology-based descriptive case series of diagnostic tumor submissions from dogs and cats, and a cross-sectional questionnaire survey of veterinarians practicing in Algeria. The first component characterized tumors reaching histopathological confirmation, whereas the second examined practitioner-reported tumor patterns, perceived diagnostic-resource availability, and self-reported preparedness.

**Results:**

The histopathology-based case series comprised 86 tumor submissions from 81 animals accrued between March 2022 and March 2025. Mammary tumors were the largest category (43/86; 50.0%), followed by cutaneous/subcutaneous tumors (20/86; 23.3%); 80 tumors (93.0%) were classified as malignant.

Among 162 questionnaire responses collected between October 2024 and May 2025, 111 respondents selected “dogs and cats” as their main animal group. Mammary tumors were reported by 109 respondents (98.2%), cutaneous/subcutaneous tumors by 79 (71.2%), and canine transmissible venereal tumor (CTVT) by 54 (48.6%); multiple categories could be reported. The most frequent self-reported preparedness rating was “Moderately prepared” (52/111; 46.8%), while 99 of 110 respondents with valid data (90.0%) rated diagnostic resources as either “Insufficient” or “Not at all satisfactory.”

Mammary and cutaneous/subcutaneous tumors ranked first and second in both sources, whereas the rankings of the remaining categories differed. Because the sources represented different populations, analytical units, denominators, and diagnostic pathways, the comparison was restricted to an exploratory descriptive assessment and was not interpreted as evidence of differences in population-level tumor occurrence.

**Conclusions:**

This study provides an initial structured account of companion-animal tumor documentation in Algeria. The partial agreement between the two sources, together with unfavorable diagnostic-availability ratings, suggests potential gaps between clinical recognition, histopathological confirmation, and systematic recording. Standardized submission and coding procedures, improved specimen-transport and formal pathology-referral pathways, broader geographic participation, and progressive development of companion-animal cancer registration are needed to strengthen the national evidence base.

**Supplementary Information:**

The online version contains supplementary material available at https://doi.org/10.1186/s12917-026-05856-3.

## Background

Large diagnostic datasets and cancer registries show that the distribution of spontaneous tumors in dogs and cats varies by species, age, sex, anatomical site, biological behavior, and histological classification [1–4]. Histopathological examination remains central to the diagnosis and classification of many solid tumors in companion animals [5]. When collected using standardized variables and diagnostic terminology, histopathology records can support descriptive comparisons and the progressive development of veterinary cancer surveillance [1, 6].

However, pathology-based datasets arise from a selected diagnostic pathway and do not necessarily represent population-level tumor occurrence [7, 8]. Entry into such datasets depends on whether a suspected tumor is clinically recognized, sampled, and submitted for diagnosis, processes that are influenced by access to veterinary and pathology services, geographic coverage, and submission practices [7, 8]. The geographic catchment of a diagnostic service and its inclusion criteria can therefore substantially influence the tumor profile observed among submitted specimens [7, 8]. The veterinary cancer-registration literature has also identified inconsistent diagnostic criteria, limited standardization, and the absence of centralized databases as important barriers to comparisons across studies and settings [6, 9]. Consequently, without clearly defined source populations and appropriate denominators, pathology-submission data should be interpreted as distributions among submitted tumors rather than estimates of incidence, prevalence, or population-level risk. Recent hospital-based analyses using the full attending population as the denominator further illustrate how explicitly defined clinical source populations can support estimation of cancer proportions among hospital attendees [10].

International studies frequently identify mammary and cutaneous/subcutaneous tumors among the prominent categories recorded in dogs and cats, although their relative distributions vary according to species, study population, diagnostic pathway, and inclusion criteria [1–4, 11]. In North Africa, a prospective Moroccan study of 250 canine and feline tumors documented setting-specific patterns, including a high representation of canine transmissible venereal tumor (CTVT), whereas squamous cell carcinoma predominated among cats [12]. Such variation supports the value of country-specific tumor documentation while demonstrating why findings from different diagnostic settings cannot be interpreted as directly comparable estimates of tumor occurrence [1–4, 12].

Published Algerian evidence on companion-animal tumors remains largely restricted to particular tumor types and geographically limited samples [13–15]. A study of 215 female dogs from four northeastern Algerian provinces described the occurrence and histopathological characteristics of canine mammary tumors [13]. Subsequent work examined mammary tumors in female cats from Constantine and Annaba [14], while a more recent study characterized canine genital tumors, with particular emphasis on CTVT, in eastern Algeria [15]. These studies provide valuable local evidence concerning specific tumors but do not characterize the broader multisystem composition of histopathologically confirmed tumors in dogs and cats or examine practitioner-reported tumor patterns, perceived availability of diagnostic resources, and self-reported preparedness within the same investigation [13–15]. To the authors’ knowledge, no national companion-animal cancer registry or standardized national tumor-reporting system has been documented for Algeria in the current veterinary cancer-registration literature [6].

In this context, the two data sources address related but distinct questions. Histopathology records characterize tumors reaching pathological confirmation, whereas a practitioner survey can capture tumor categories encountered clinically, perceived availability of diagnostic resources, and self-reported preparedness. Examining the sources separately within the same investigation may therefore identify points of convergence and potential gaps across clinical recognition, diagnostic submission, and systematic documentation.

Accordingly, this study aimed to: first, characterize histopathologically confirmed tumor submissions from dogs and cats according to species, sex, age, broad tumor category, biological behavior, morphologic category, and grouped histopathological diagnosis; second, describe practitioner-reported tumor patterns, perceived availability of diagnostic resources, and self-reported preparedness among veterinarians treating companion animals in Algeria; and third, explore broad concordances and discrepancies between the two sources at an aggregated descriptive level. The study was not designed to estimate tumor incidence, prevalence, or population-level risk.

## Methods

### Overall study design

This dual-source descriptive study comprised two independently analyzed components: a histopathology-based case series of diagnostic tumor submissions from dogs and cats received between March 2022 and March 2025; and a cross-sectional questionnaire survey of veterinarians practicing in Algeria, conducted between October 2024 and May 2025.

The histopathology component characterized tumors reaching histopathological confirmation through a diagnostic-submission pathway, whereas the questionnaire component characterized practitioner-reported tumor patterns, perceived availability of diagnostic resources, and self-reported preparedness. The two components were analyzed separately because they differed in their populations, units of analysis, sampling pathways, geographic coverage, and potential sources of bias.

The datasets were not linked at the animal, veterinarian, or practice level. Some veterinarians who submitted specimens may also have participated in the questionnaire; however, because the survey was anonymous and the datasets were unlinked, any overlap could not be identified or quantified. Cross-source comparison was therefore restricted to an exploratory descriptive assessment of broad tumor-category rankings after terminology harmonization. No direct statistical comparison between the sources was performed.

### Histopathology-based case series

From March 2022 to March 2025, diagnostic tumor submissions from dogs and cats were received. The histopathological processing and evaluation were performed at the Anatomic Pathology Laboratory, Institut des Sciences Vétérinaires El-Khroub, Université Constantine 1 Frères Mentouri. The laboratory provided study-associated diagnostic histopathology and was not part of a routine national veterinary pathology submission network. Eligible submissions originated from Constantine, Algiers, Setif, Annaba, Tebessa, and Boumerdes.

Biopsies and surgical excisions were performed by the submitting veterinarians for clinical purposes before submission. The study did not determine or modify biopsy, surgical, treatment, or other clinical-management decisions, and no additional tissue was collected for research purposes. Specimens were placed in formalin by the submitting veterinarians; when transport assistance was required, formalin-fixed specimens were collected directly from the veterinary practices. Histopathological examination and diagnostic reporting were provided free of charge for the included submissions, irrespective of their geographic origin.

The analytical series comprised all retrievable diagnostic submissions received during the study period that originated from a dog or cat, showed histologically confirmed neoplasia, and permitted assignment of a reportable histopathological diagnosis and biological behavior. Cases were not sampled from the eligible tumor submissions.

Qualified subtype labels, such as “favored,” were retained when neoplasia and broad biological behavior were established but the exact line of differentiation could not be determined confidently using hematoxylin and eosin sections alone. Submissions from other species, specimens received outside the study period, non-neoplastic lesions, non-diagnostic or inadequately preserved specimens, cases for which neoplasia or biological behavior could not be established, and duplicate records referring to the same specimen were excluded. Because a complete screening log of all specimens assessed and excluded was not available, the numbers initially screened and excluded could not be reconstructed.

The unit of analysis was the separately submitted tumor specimen rather than the individual animal. A separately submitted recurrent tumor was retained as an additional tumor submission. A histopathologically distinct second primary tumor arising in an animal already represented in the series was also retained as a separate submission. Consequently, the numbers of tumor submissions and individual animals were reported separately in the Results.

For each eligible submission, the available recorded information comprised species, sex, age, geographic origin, anatomical location, histopathological diagnosis, and biological behavior. Specimens were fixed in 10% formalin, processed routinely, embedded in paraffin, sectioned at 5 μm, and stained with hematoxylin and eosin. Diagnoses were established using standard histomorphological criteria, including tissue architecture, cellular morphology, stromal characteristics, evidence of invasion, and other tumor-specific features, with reference to *Tumors in Domestic Animals* [5].

Tumors were classified as benign or malignant according to their confirmed histopathological diagnosis and morphological characteristics. Biological behavior was assigned from the final histopathological diagnosis and histomorphological features. Diagnoses designated as carcinomas, adenocarcinomas, sarcomas, lymphomas, CTVT, melanomas, or other explicitly malignant neoplasms were coded as malignant. Adenomas, trichoblastomas, melanocytomas, and other explicitly benign neoplasms were coded as benign. No intermediate or uncertain-behavior category was used. Given their variable biological behavior, the two canine cutaneous mast cell tumors were assigned a priori to the malignant category for this analysis [5]; neither was reclassified as benign. Cases for which biological behavior could not be established were excluded.

Dedicated veterinary immunohistochemistry and other ancillary pathology techniques were not routinely available. The analytical series was therefore restricted to tumors for which neoplasia, a reportable diagnosis, and biological behavior could be established through routine histopathological examination.

### Cross-sectional questionnaire survey

The target population comprised veterinarians practicing in Algeria, regardless of their principal animal sector or type of practice. For the present companion-animal analyses, the eligible subset comprised respondents who selected “dogs and cats” when asked which animals they mainly treated. The numbers of total responses and eligible respondents are reported in the Results section.

A non-probability convenience-sampling approach with peer forwarding was used. The online questionnaire was circulated by email and through veterinary Facebook groups, LinkedIn, and WhatsApp, and recipients could forward it to other practicing veterinarians. Paper questionnaires were distributed during visits to veterinary practices and at professional meetings and conferences. Participation was voluntary and anonymous; names, email addresses, and other direct contact identifiers were neither collected nor recorded.

An official government communication estimated that Algeria had approximately 20,000 veterinarians across all professional sectors in 2022 [16]. However, no publicly available national sampling frame identified veterinarians who were actively practicing during the survey period or specifically treating companion animals. Because recruitment occurred through open professional networks, paper distribution, and peer forwarding, the number of unique eligible veterinarians reached could not be determined; consequently, a conventional response rate could not be calculated. No a priori sample-size calculation was performed because the survey was exploratory and descriptive, with no prespecified primary hypothesis, effect size, or precision target. Recruitment therefore sought the maximum feasible participation during the predefined survey period, and the resulting convenience sample was not considered nationally representative.

The structured questionnaire covered five domains: veterinarian and practice characteristics; frequency and types of tumors encountered; diagnostic and therapeutic practices; perceptions and continuing education; and challenges in tumor management. The original French-language questionnaire and its English translation are provided in Additional file 2.

Professional experience was recorded in years. The reported frequency of diagnosing tumors in daily practice was assessed using five ordered categories: “Very rarely,” “Rarely,” “Sometimes,” “Frequently,” and “Very frequently.” These were qualitative response categories; the questionnaire did not assign them fixed numbers of cases or specific time intervals. For ordinal analysis, they were coded from 1 to 5, with higher values indicating greater reported tumor-diagnosis frequency.

Perceived availability of diagnostic resources was assessed using four ordered categories: “Very satisfactory,” “Satisfactory,” “Insufficient,” and “Not at all satisfactory.” Self-reported preparedness to diagnose and treat tumors was assessed using five ordered categories: “Very well prepared,” “Well prepared,” “Fairly prepared,” “Moderately prepared,” and “Not well prepared.” These variables were coded so that higher values represented greater perceived diagnostic availability and greater self-reported preparedness, respectively. The original response labels were retained when presenting the descriptive results.

The tumor-category questions allowed multiple responses. Responses from the combined dog-and-cat question and the species-specific questions were harmonized into six categories for descriptive reporting: mammary, cutaneous/subcutaneous, canine transmissible venereal tumor (CTVT), bone, digestive, and respiratory tumors. French and English wording variants and clearly equivalent free-text responses were standardized before coding. Each respondent was counted no more than once within a given tumor category, even if that category was selected or mentioned in more than one questionnaire item; consequently, categories were not mutually exclusive.

For CTVT, only explicit references to CTVT, TVT, or Sticker tumor/sarcoma in the dog-specific response field were coded as CTVT. Generic references to genital-tract tumors were not assumed to represent CTVT, and responses entered in the feline-specific field were not classified as CTVT. Lymphoma responses were not included in the six-category cross-source comparison because lymphoma is a diagnostic or morphologic category and the questionnaire did not provide sufficient anatomical information to map these responses to a corresponding histopathology tumor category.

Missing and uncodable responses were not imputed. They were excluded only from the analysis of the corresponding item, and percentages were calculated using the number of valid responses for that item.

### Data processing and harmonization

The histopathology and questionnaire datasets were cleaned and coded independently. Species terminology was standardized as canine or feline, sex as female or male, and geographic origin as the corresponding Algerian wilaya. French and English variants, spelling differences, and minor formatting inconsistencies were harmonized before analysis.

Each histopathology submission was assigned to one mutually exclusive broad tumor category according to its anatomical location and definitive diagnosis: mammary, cutaneous/subcutaneous, CTVT, digestive, respiratory, or bone. These categories were selected to permit an aggregated descriptive comparison with the corresponding practitioner-reported categories. The term “tumor category” was used instead of “tumor system” because CTVT represents a specific diagnosis rather than an anatomical system.

Histopathological diagnoses were also classified into four broad morphologic categories: epithelial/complex epithelial, round-cell, mesenchymal, and melanocytic tumors. The epithelial/complex epithelial category included carcinomas, adenocarcinomas, adenomas, and complex epithelial mammary tumors. The round-cell category included lymphoma, mast cell tumor, and CTVT. Sarcomas and other tumors of mesenchymal origin were classified as mesenchymal, whereas melanomas and other melanocytic neoplasms were classified as melanocytic.

For presentation in Table 3, histopathological diagnoses were additionally consolidated into clinically interpretable diagnostic groups to avoid fragmentation into numerous small categories. Diagnoses that could not be included in one of the principal groups, including rare single-case diagnoses, were combined under “Other histopathological diagnoses.” The complete ungrouped list of histopathological diagnoses is provided in Additional file 1: Table S1.

Questionnaire percentages were calculated using respondents as the denominator, whereas histopathology percentages were calculated using separately submitted tumors. Percentages were calculated independently within each source and were not pooled.

For geographic presentation, the respondent’s principal practice wilaya (region) was used for the questionnaire dataset, whereas the recorded origin of the animal or submitting practice was used for the histopathology dataset. For figure readability, the ten wilayas (regions) contributing the largest combined numbers of records were displayed individually, and the remaining wilayas were grouped as “Other wilayas.” This grouping was used only for visualization; no observations were excluded, and the complete wilaya-specific distribution is provided in Additional file 1: Table S3.

### Statistical analysis

All analyses were performed using R version 4.5.3. Categorical variables were summarized as numbers and percentages. Age and years of professional experience were summarized using the median, interquartile range, and minimum–maximum values.

Within the histopathology dataset, associations between categorical variables were evaluated using Fisher’s exact method. For sparse contingency tables with more than two categories, Monte Carlo p-values were estimated using 10,000 replicates. Age was compared between two groups using the Wilcoxon rank-sum method and across more than two groups using the Kruskal–Wallis method.

Within the questionnaire dataset, associations among reported tumor-diagnosis frequency, perceived diagnostic availability, self-reported preparedness, and years of professional experience were assessed using Spearman’s rank correlation. Correlations were calculated using pairwise complete observations, and the number of respondents included in each analysis was reported. Because tied ordinal responses were present, asymptotic p-values were used.

Geographic distributions were analyzed descriptively. No inferential comparison was performed between questionnaire responses and histopathology submissions because the two sources differed in their sampling pathways, units of analysis, denominators, and geographic coverage.

All statistical analyses were exploratory. Where applicable, tests were two-sided, and a nominal p-value below 0.05 was used to identify results warranting descriptive attention. No adjustment for multiple comparisons was applied; therefore, p-values were interpreted as exploratory indicators rather than confirmatory evidence. No multivariable modelling was undertaken because of the sample size and sparse outcome categories.

### Generative AI use

During the preparation of this manuscript, the authors used AI tools for language editing, grammar refinement, improvement of readability, and assistance with R code checking and troubleshooting. The tool was not used to generate study data, perform independent analyses, produce or alter results, create figures, interpret findings, or formulate scientific conclusions. All analyses, results, interpretations, and final manuscript content were reviewed, verified, and approved by the authors, who take full responsibility for the integrity and accuracy of the work.

## Results

### Histopathology-based case series

The final histopathology dataset comprised 86 eligible tumor submissions originating from 81 individual animals, including four recurrent tumor submissions and one histopathologically distinct second primary tumor. Dogs accounted for 64 submissions (74.4%) and cats for 22 (25.6%), while 55 submissions (64.0%) originated from females and 31 (36.0%) from males. The median age was 8 years (IQR, 6–9; range, 1–14 years) (Table 1).

**Table 1:** Overall characteristics of the histopathology-based case series

Study: Histopathology-based case series		
Characteristic	Category/statistic	Result
Analytical dataset	Total tumor submissions	86
	Individual animals represented	81
	Recurrent tumor submissions	4
	Second primary tumor submission	1
Species	Canine	64 (74.4%)
	Feline	22 (25.6%)
Sex	Female	55 (64.0%)
	Male	31 (36.0%)
Age, years	Median (IQR; minimum–maximum)	8 (6–9; 1–14)
Biological behavior	Malignant	80 (93.0%)
	Benign	6 (7.0%)
Morphologic category	Epithelial/complex epithelial	52 (60.5%)
	Round-cell	17 (19.8%)
	Mesenchymal	13 (15.1%)
	Melanocytic	4 (4.7%)

Values are *n*(%) unless otherwise indicated. Percentages for species, sex, biological behavior, and morphologic category use the 86 tumor submissions as the denominator. The 86 submissions originated from 81 individual animals. Four submissions represented recurrent tumors, and one represented a histopathologically distinct second primary tumor in an animal already represented in the series. Each separately submitted tumor constituted one analytical case

*IQR* interquartile range

Mammary tumors constituted the largest category, accounting for 43 submissions (50.0%), followed by cutaneous/subcutaneous tumors with 20 (23.3%). CTVT and digestive tumors each accounted for ten submissions (11.6%), whereas respiratory and bone tumors accounted for two (2.3%) and one (1.2%), respectively. All mammary tumors originated from females, while all CTVT submissions and the single bone tumor originated from dogs. Species, sex, biological behavior, and age distributions within each tumor category are presented in Table 2.

**Table 2:** Distribution of histopathologically confirmed tumor submissions by tumor category, species, sex, biological behavior, and age

Tumor category	Total, *n* (%)	Species, *n* (% within category)	Sex, *n* (% within category)	Biological behavior, *n* (% within category)	Age, median (minimum–maximum), years
Mammary	43 (50.0%)	Canine: 29 (67.4%)	Female: 43 (100%)	Malignant: 41 (95.3%)	8 (5–14)
		Feline: 14 (32.6%)	Male: 0	Benign: 2 (4.7%)	
Cutaneous/ subcutaneous	20 (23.3%)	Canine: 16 (80.0%)	Female: 7 (35.0%)	Malignant: 16 (80.0%)	8 (5–12)
		Feline: 4 (20.0%)	Male: 13 (65.0%)	Benign: 4 (20.0%)	
CTVT	10 (11.6%)	Canine: 10 (100%)	Female: 2 (20.0%)	Malignant: 10 (100%)	8 (5–12)
		Feline: 0	Male: 8 (80.0%)	Benign: 0	
Digestive	10 (11.6%)	Canine: 7 (70.0%)	Female: 1 (10.0%)	Malignant: 10 (100%)	6.5 (1–9)
		Feline: 3 (30.0%)	Male: 9 (90.0%)	Benign: 0	
Respiratory	2 (2.3%)	Canine: 1 (50.0%)	Female: 2 (100%)	Malignant: 2 (100%)	7.5 (7–8)
		Feline: 1 (50.0%)	Male: 0	Benign: 0	
Bone	1 (1.2%)	Canine: 1 (100%)	Female: 0	Malignant: 1 (100%)	6
		Feline: 0	Male: 1 (100%)	Benign: 0	

The analytical unit was the tumor submission (*n* = 86). Percentages in the “Total” column use all 86 submissions as the denominator. Species, sex, and biological-behavior percentages are calculated within each tumor-category row. Age is reported as median (minimum–maximum); the single observed age is shown for the bone-tumor category

Malignant tumors accounted for 80 submissions (93.0%). Epithelial/complex epithelial tumors constituted the predominant morphologic category (52/86; 60.5%), and mammary carcinoma was the largest grouped histopathological diagnosis (36/86; 41.9%) (Tables 1 and 3). The complete ungrouped distribution of histopathological diagnoses is provided in Additional file 1: Table S1.

**Table 3:** Distribution of grouped histopathological diagnoses among 86 tumor submissions

Grouped histopathological diagnosis	*n* (%)
Mammary carcinoma	36 (41.9)
CTVT	10 (11.6)
Other histopathological diagnoses	10 (11.6)
Cutaneous/adnexal epithelial tumor	8 (9.3)
Mammary fibrosarcoma/spindle-cell sarcoma	5 (5.8)
Cutaneous/subcutaneous soft-tissue sarcoma	4 (4.7)
Melanocytic tumor	4 (4.7)
Lymphoma	3 (3.5)
Anal sac apocrine gland adenocarcinoma	2 (2.3)
Benign mammary epithelial/complex lesions	2 (2.3)
Mast cell tumor	2 (2.3)

Values are* n *(%), using the 86 tumor submissions as the denominator. Diagnostic groups represented by single cases and residual minor diagnoses were combined as “Other histopathological diagnoses.” The complete ungrouped diagnostic distribution is provided in Supplementary Table S1

Sex distribution differed across tumor categories (Monte Carlo Fisher’s exact *p* < 0.001), and sex was associated with the mammary versus non-mammary classification (Fisher’s exact *p* < 0.001). Median age was 8 years (IQR, 7–10) among females and 7 years (IQR, 6–8) among males (Wilcoxon *p* = 0.012). None of the other evaluated associations met the nominal 0.05 threshold (Table 4).

**Table 4:** Exploratory associations within the histopathology and questionnaire datasets. Exploratory analyses in the histopathology-based case series

Analysis	Statistical method	*p* value
Species and tumor-category distribution	Fisher exact, simulated p value	0.223
Species and biological behavior	Fisher exact	0.331
Sex and tumor-category distribution	Fisher exact, simulated p value	< 0.001
Mammary versus non-mammary category by sex	Fisher exact	< 0.001
Age by species	Wilcoxon rank-sum	0.363
Age by sex	Wilcoxon rank-sum	0.012
Age across tumor categories	Kruskal–Wallis	0.149
Species and morphologic category	Fisher exact, simulated p value	0.078

Presents categorical associations evaluated using Fisher’s exact method, with Monte Carlo *p*-values based on 10,000 replicates for sparse contingency tables, and age comparisons evaluated using Wilcoxon rank-sum or Kruskal–Wallis methods

### Cross-sectional questionnaire survey

A total of 162 questionnaire responses were received. Of these, 111 respondents selected dogs and cats as the animals they mainly treated and constituted the companion-animal analytical subset. Among the 110 respondents with available professional-experience data, the median duration of practice was 5 years (IQR, 3–9; range, 0–40 years) (Table 5).

**Table 5:** Questionnaire sample, professional experience, reported tumor-case exposure, self-reported preparedness, and diagnostic-resource availability

Study: Cross-sectional questionnaire survey		
Characteristic	Category/statistic	Result
Analytical dataset	All questionnaire respondents	162
	Companion-animal analytical subset	111 (68.5%)
Professional experience, years	Median (IQR; minimum–maximum)	5 (3–9; 0–40), *n* = 110
Tumor-case exposure	Very rarely	13 (11.7%)
	Rarely	15 (13.5%)
	Sometimes	63 (56.8%)
	Frequently	18 (16.2%)
	Very frequently	2 (1.8%)
Self-reported preparedness	Not well prepared	14 (12.6%)
	Moderately prepared	52 (46.8%)
	Fairly prepared	41 (36.9%)
	Well prepared	2 (1.8%)
	Very well prepared	2 (1.8%)
Diagnostic-resource availability	Very satisfactory	0 (0.0%)
	Satisfactory	11 (10.0%)
	Insufficient	66 (60.0%)
	Not at all satisfactory	33 (30.0%)
	Missing/uncoded	1

Values are *n*(%) unless otherwise indicated. The companion-animal analytical subset comprised respondents who selected “dogs and cats” when asked which animals they mainly treated. Percentages for tumor-case exposure and preparedness use 111 respondents as the denominator. Percentages for diagnostic-resource availability use the 110 valid responses as the denominator; one missing or uncoded response was excluded. The response-category labels are reproduced as presented in the questionnaire

*IQR *interquartile range

“Sometimes” was the most frequently selected tumor-case exposure category (63/111; 56.8%). Self-reported preparedness was concentrated in the “Moderately prepared” (52/111; 46.8%) and “Fairly prepared” (41/111; 36.9%) categories. Diagnostic-resource availability was rated as “Insufficient” by 66 of 110 respondents (60.0%) and “Not at all satisfactory” by 33 (30.0%). Thus, 90.0% of respondents with valid data selected one of the two unfavorable diagnostic-availability categories. Complete response distributions are presented in Table 5.

**Fig. 1:**
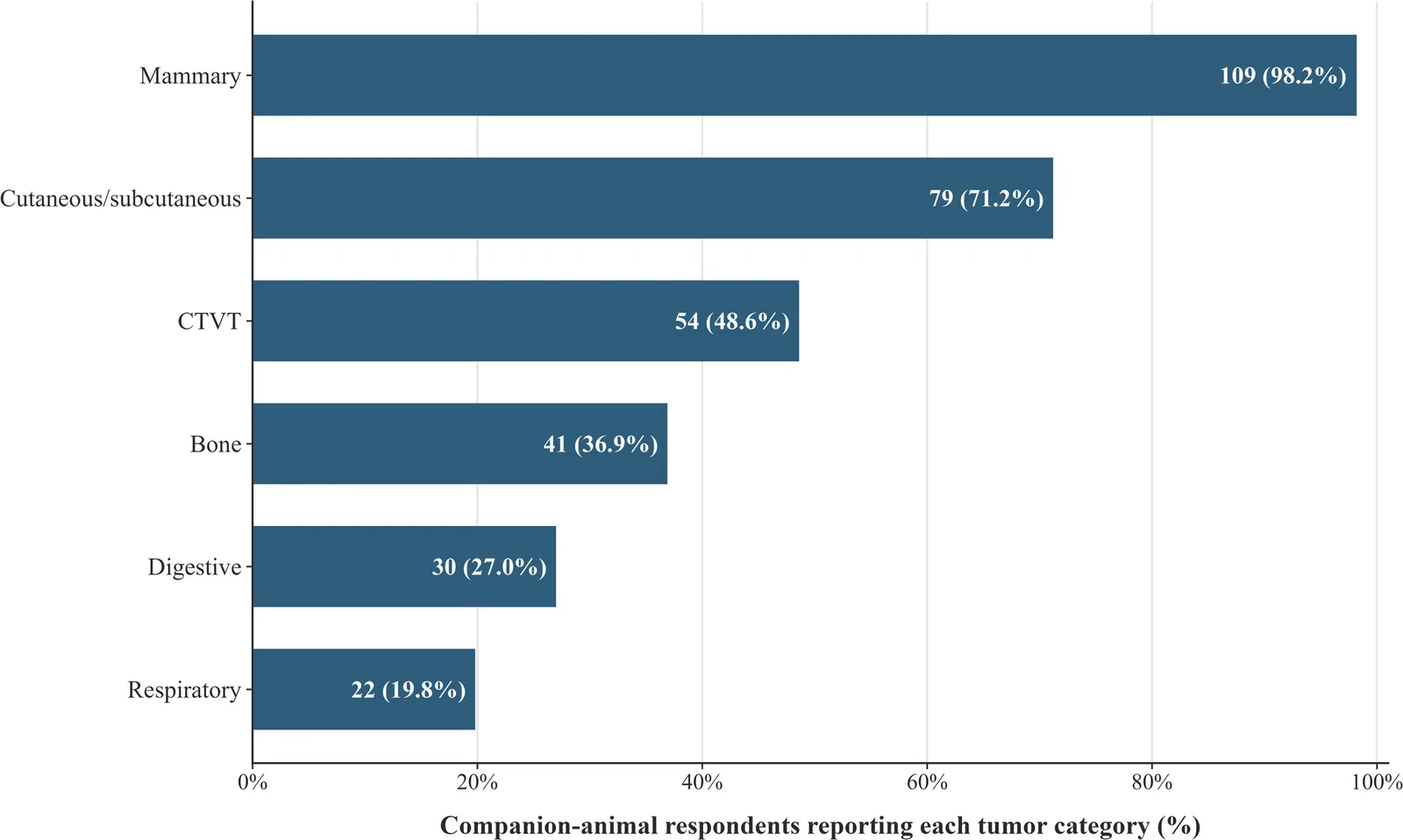
Practitioner-reported tumor categories in the companion-animal questionnaire subset in Algeria. Bars show the number and percentage of the 111 companion-animal respondents who reported encountering each tumor category in practice. Respondents could report multiple categories; therefore, percentages use 111 as the denominator and are not mutually exclusive. These findings represent practitioner-reported experience and should not be interpreted as population-level incidence or prevalence

Because multiple tumor categories could be reported by each respondent, the categories were not mutually exclusive. Mammary tumors were reported by 109 respondents (98.2%), followed by cutaneous/subcutaneous tumors by 79 (71.2%) and CTVT by 54 (48.6%). The complete distribution is presented in Fig. 1 and Additional file 1: Table S2.

Within the questionnaire dataset, reported tumor-diagnosis frequency was positively correlated with self-reported preparedness and perceived diagnostic availability. Perceived diagnostic availability was also positively correlated with self-reported preparedness. Professional experience was not correlated with tumor-diagnosis frequency, preparedness, or perceived diagnostic availability (Table 6).

**Table 6:** Exploratory associations within the histopathology and questionnaire datasets. Spearman rank correlations among questionnaire variables

Association	*n*	Spearman’s ρ	*p* value
Reported tumor-diagnosis frequency and self-reported preparedness	111	0.383	< 0.001
Reported tumor-diagnosis frequency and perceived diagnostic availability	110	0.283	0.003
Perceived diagnostic availability and self-reported preparedness	110	0.355	< 0.001
Professional experience and reported tumor-diagnosis frequency	110	0.027	0.777
Professional experience and self-reported preparedness	110	−0.035	0.716
Professional experience and perceived diagnostic availability	109	0.046	0.636

Presents Spearman rank correlations among reported tumor-diagnosis frequency, perceived diagnostic availability, self-reported preparedness, and professional experience. Sample sizes reflect pairwise complete observations. Reported *p*-values are unadjusted and should be interpreted as exploratory

### Geographic coverage of the two data sources

**Fig. 2:**
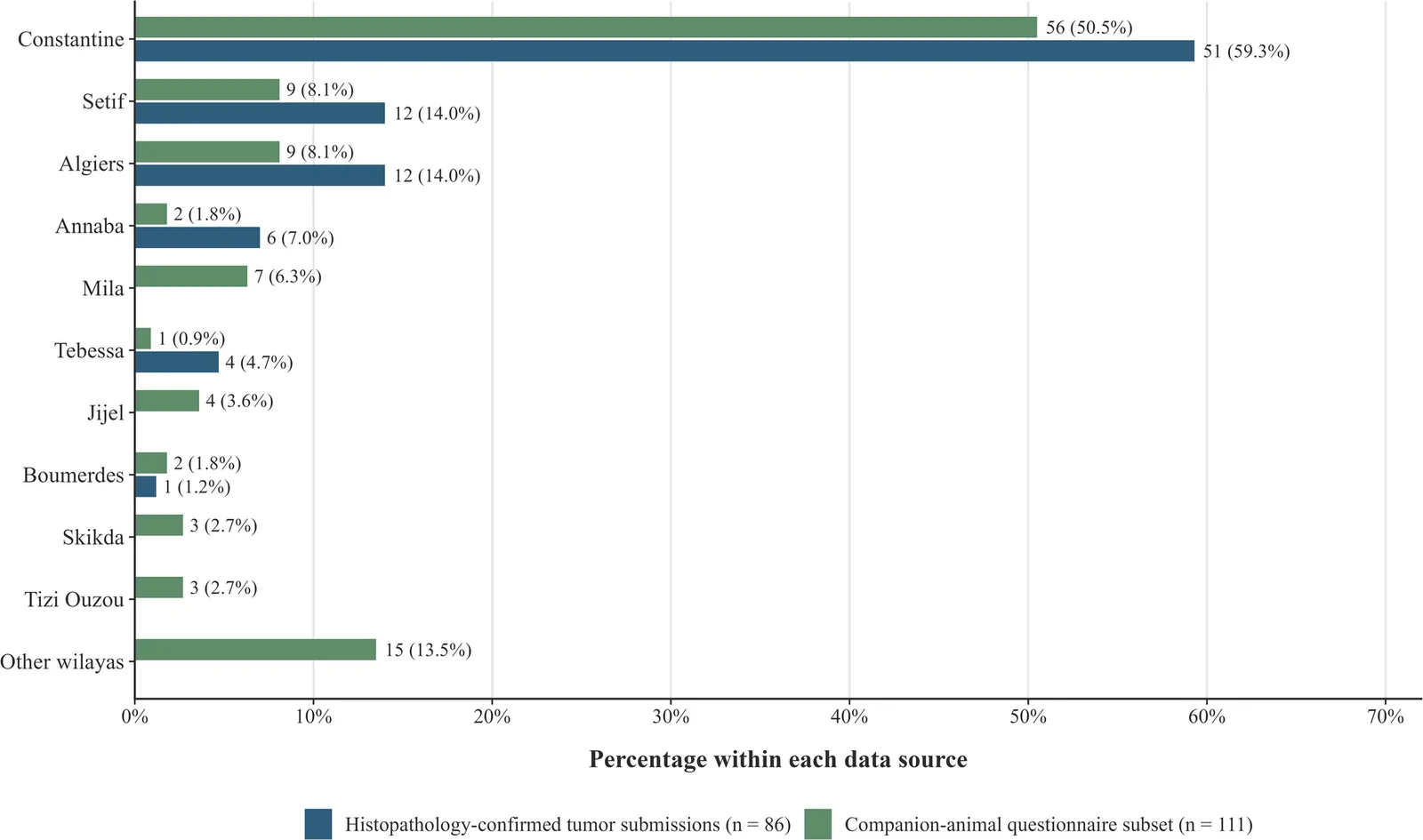
Geographic coverage of histopathology-confirmed tumor submissions and questionnaire respondents across Algerian wilayas/regions. Bars show the number and within-source percentage of 86 histopathology-confirmed tumor submissions and 111 respondents included in the companion-animal questionnaire subset. To maintain figure readability, the ten wilayas with the highest combined number of records across the two data sources are displayed individually. The remaining 11 wilayas, which contributed 15 questionnaire responses and no histopathology submissions, were combined as “Other wilayas”; no observations were excluded. Percentages were calculated separately for each data source. The figure characterizes geographic coverage and should not be interpreted as regional tumor frequency or population representativeness. Complete wilaya-specific counts are provided in Additional file 1: Table S3

The histopathology-based case series included submissions from six Algerian wilayas, whereas the questionnaire subset included respondents from 21 wilayas. Constantine was the largest contributor to both sources, accounting for 51 of 86 histopathology submissions (59.3%) and 56 of 111 questionnaire respondents (50.5%). Figure 2 summarizes the principal geographic distribution, and complete wilaya-specific counts are provided in Additional file 1: Table S3. These distributions describe the histopathology-submission catchment and questionnaire coverage and should not be interpreted as geographic differences in tumor occurrence.

### Exploratory descriptive comparison of the two data sources

Histopathology percentages represented the distribution of 86 mutually exclusive tumor submissions, whereas questionnaire percentages represented the proportions of 111 respondents reporting each category, with multiple categories permitted per respondent. The numerical percentages were therefore not directly compared; the analysis was restricted to broad category rankings, and no cross-source inferential analysis was performed.

Mammary tumors ranked first and cutaneous/subcutaneous tumors second in both sources. CTVT ranked third among practitioner-reported categories and joint third with digestive tumors among histopathology submissions. Bone tumors ranked fourth in the questionnaire but were represented by only one histopathology submission, whereas digestive tumors occupied a higher relative rank among histopathology submissions. Respiratory tumors were among the least prominent categories in both sources.

## Discussion

### Principal findings and interpretation of the study design

This study provides an initial structured description of companion-animal tumor documentation in Algeria using two independently analyzed data sources. Mammary and cutaneous/subcutaneous tumors were the two highest-ranked categories in both sources, whereas the rankings of the remaining categories differed. The two sources are complementary but not equivalent: they describe different stages of tumor recognition and documentation rather than providing two estimates of the same epidemiological measure. Accordingly, all cross-source interpretation is exploratory and descriptive and is limited to broad patterns of convergence and divergence.

The findings represent patterns of clinical and diagnostic visibility rather than estimates of population-level tumor burden. Histopathology submissions depend on clinical recognition, sampling decisions, access to diagnostic services, and submission pathways, whereas questionnaire responses reflect practitioner experience and perceptions within a non-probability sample. Neither source included the population denominators required to estimate incidence, prevalence, or risk. Their combined value lies in identifying potential gaps between clinical recognition, pathological confirmation, and systematic recording. This is particularly relevant in the absence of an established national companion-animal cancer registry, where standardized clinical and pathological data are needed to support the progressive development of cancer surveillance [6–9].

### Interpretation of the histopathology-submitted tumor distribution

Mammary tumors constituted the largest category among the histopathology submissions, and mammary carcinoma was the leading grouped diagnosis. This pattern is consistent with the recognized importance of mammary neoplasia in female dogs and cats and with pathology-based studies in which mammary tumors constitute a substantial component of submitted material [2, 12, 17–19]. Nevertheless, their prominence in this series should not be interpreted as evidence that mammary tumors are the most common tumors in the wider Algerian dog and cat population. Mammary masses are externally visible, commonly managed surgically, and readily sampled, which may increase their probability of histopathological submission relative to less accessible tumors.

The observed associations involving sex and age should also be interpreted in relation to the composition of the submitted series. All mammary-tumor submissions originated from females, making the association between sex and tumor category largely attributable to the concentration of mammary tumors within the female subset. Similarly, the older age distribution among females may partly reflect the age profile of animals submitted with mammary tumors. Although the predominance of tumors in mature animals is consistent with established age-related patterns in companion-animal oncology [20, 21], the absence of source-population denominators, reproductive-status information, and an unaffected comparison group prevents estimation of independent sex- or age-associated risk.

The high proportion of malignant diagnoses represents malignant enrichment within submitted material rather than a population-level malignancy rate. Clinically concerning, rapidly growing, invasive, recurrent, ulcerated, or diagnostically uncertain lesions may be more likely to undergo biopsy or excision and subsequent histopathological examination. Conversely, small or clinically benign-appearing lesions may remain unsampled, be removed without histopathological examination, or remain undocumented. Similar selection processes affect pathology-based veterinary tumor datasets and can substantially influence their apparent biological-behavior distribution [4, 7, 8].

### Diagnostic availability, preparedness, and potential under-documentation

The predominance of unfavorable diagnostic-availability ratings provides relevant context for understanding why clinically suspected tumors may not consistently progress to pathological confirmation. These ratings reflect practitioner perceptions rather than an objective inventory of national diagnostic capacity, but they indicate that access to appropriate resources was widely regarded as constrained within the questionnaire sample. The concentration of self-reported preparedness in intermediate categories further suggests that diagnostic access and professional confidence may represent related components of the same clinical pathway.

The modest positive correlations among reported tumor-diagnosis frequency, perceived diagnostic availability, and self-reported preparedness are compatible with interrelated effects of case exposure, access to diagnostic resources, and professional confidence. However, their direction cannot be determined from cross-sectional self-reported data, and they should not be interpreted causally. Similarly, the absence of a correlation with years of professional experience does not establish that professional experience is unrelated to oncology preparedness, particularly given the non-probability sample and the exploratory nature of the analyses.

Publicly available information documents a dedicated veterinary anatomic and cytologic pathology laboratory in Algeria [22]. However, the presence of an individual service does not establish a routinely accessible and coordinated national submission pathway for companion-animal specimens. Because the questionnaire did not ask respondents to identify the laboratories or referral pathways they used, the availability, geographic accessibility, and consistency of these diagnostic pathways could not be evaluated from the present data.

Within the diagnostic service used for the present study, dedicated veterinary ancillary pathology techniques, particularly immunohistochemistry, were not routinely available. Access to such techniques through human medical facilities was not systematic and could be limited by cost, availability, and prioritization of human diagnostic needs. These constraints may restrict diagnostic refinement for poorly differentiated or morphologically ambiguous tumors, for which ancillary techniques can support classification [5]. Nevertheless, all cases retained in the histopathology series had histologically confirmed neoplasia, a reportable histopathological diagnosis, and assignable biological behavior based on routine histomorphological examination. More broadly, restricted access to specialist pathology services, specimen transportation, ancillary testing, and continuing education may contribute to potential underdiagnosis or under-documentation [8]. This does not demonstrate that tumors were absent from clinical practice; rather, it suggests that some suspected cases may not progress through the complete pathway from recognition to sampling, pathological confirmation, and standardized recording [6, 8].

### Cross-source discrepancies, submission pathways, and geographic accessibility

The shared prominence of mammary and cutaneous/subcutaneous tumors may partly reflect their clinical visibility and accessibility for examination, biopsy, or surgical excision. By contrast, CTVT and bone tumors occupied higher relative positions in practitioner-reported experience than among histopathology submissions, whereas digestive tumors occupied a higher relative position in the submitted series. These ranking differences should not be interpreted as differences in tumor occurrence. They more plausibly reflect variation in clinical recognition, diagnostic work-up, biopsy feasibility, and the probability that a suspected tumor progresses to histopathological examination.

CTVT has distinctive clinical characteristics and may be recognized and managed without routine histopathological confirmation in some cases [23]. Bone tumors may generate substantial clinical concern but can require imaging, invasive sampling, or referral before a definitive histopathological diagnosis is obtained. Conversely, digestive tumors that proceed to surgery or biopsy are likely to generate tissue for histopathological examination. Although pathology-supported datasets are valuable for characterizing digestive tumors, their distributions remain dependent on diagnostic and submission pathways [7, 8, 24]. These explanations are plausible interpretations of the observed rankings rather than mechanisms directly measured in the present study.

Although histopathological examination and reporting were provided free of charge, this did not remove costs incurred before and after submission. Veterinary consultation, imaging, anesthesia, biopsy or surgical excision, formalin fixation, specimen transportation, and subsequent treatment may still create financial and logistical barriers. Owner willingness or ability to continue diagnostic investigation, together with the capacity of veterinarians to absorb unreimbursed costs, may therefore influence whether a clinically suspected tumor is sampled and submitted. These factors were not measured systematically and cannot be quantified in this study, but they remain potential sources of attrition along the pathway from clinical recognition to pathological documentation [7, 8].

The geographic concentration of histopathology submissions should similarly be interpreted as the catchment of the Constantine-based diagnostic service rather than as a spatial pattern of cancer susceptibility. Algeria’s geographic extent, travel distances, transportation time, specimen-shipping costs, and access to suitable submission routes may have limited contributions from more distant wilayas. Active collection of formalin-fixed specimens from some veterinary practices also influenced which submissions could reach the laboratory. Consequently, a larger number of submissions from a particular wilaya should not be interpreted as increased cancer risk, while few or no submissions should not be interpreted as a low tumor burden. Figure 2 therefore describes diagnostic access and submission flow rather than regional tumor occurrence.

### Limitations and implications for veterinary cancer surveillance

Several limitations define the scope of inference from this study. The histopathology-based case series originated from a single diagnostic service and was not derived from a defined source population. No denominator was available for the wider population of dogs and cats or for all animals presenting with suspected tumors, and the initial numbers of specimens screened and excluded were unavailable. The sample was also limited for cats and for less represented categories such as respiratory and bone tumors. Because the analytical unit was the separately submitted tumor, the findings describe tumor submissions rather than animal-level risk. Reliance on routine histomorphological examination and exclusion of non-diagnostic specimens may have limited diagnostic granularity and influenced the composition of the final series, although all retained cases met the predefined eligibility criteria for histologically confirmed neoplasia, a reportable histopathological diagnosis, and assignable biological behavior [7–9].

The questionnaire survey used non-probability convenience sampling and peer forwarding. A reliable national sampling frame and denominator were unavailable, the number of veterinarians exposed to the invitation was unknown, and a formal response rate could not be calculated. No a priori sample-size calculation was performed because the survey was exploratory, had no prespecified primary hypothesis or precision target, and sought the maximum feasible participation during the study period. These features limit the representativeness and precision of the questionnaire findings. Responses were also self-reported and may have been influenced by recall, the clinical visibility of particular tumors, individual interpretation of qualitative response categories, and uneven geographic participation.

The datasets differed in their analytical units, geographic coverage, sampling mechanisms, and collection periods. The veterinarians and wilayas represented in the questionnaire were not necessarily the same as those contributing histopathology submissions. Some veterinarians who submitted specimens may also have participated in the questionnaire, but anonymous data collection and the absence of dataset linkage prevented any overlap from being identified or quantified. Consequently, no animal-, veterinarian-, practice-, or regionally matched comparison was possible.

Despite these limitations, the study identifies practical priorities for improving companion-animal tumor documentation in Algeria. Standardized tumor-submission forms could establish a minimum dataset including species, breed, sex, age, reproductive status when available, anatomical site, clinical history, recurrence status, diagnostic method, and final histopathological diagnosis. Consistent animal and tumor identifiers would help distinguish individual animals, recurrent tumors, and separate primary tumors. Harmonized histopathological terminology and coding, improved specimen-transport pathways, broader participation across wilayas, and continuing education in biopsy, cytology, and pathology referral could strengthen case ascertainment and comparability.

A publicly accessible directory of facilities accepting veterinary specimens, standardized submission requirements, and formal referral agreements among veterinary practices, university laboratories, veterinary diagnostic services, and other pathology providers could reduce reliance on informal professional contacts. A progressive cancer-registration system could build initially on these diagnostic pathways and expand as participation and geographic coverage improve. Such a system would not immediately eliminate selection bias, but standardized recording and broader participation would improve the completeness and interpretability of future data and support gradual development from isolated diagnostic records toward coordinated companion-animal cancer surveillance [1, 6, 9].

## Conclusions

This dual-source study provides an initial structured account of companion-animal tumor documentation in Algeria. The histopathology-based case series described tumors reaching pathological confirmation, whereas the questionnaire characterized practitioner-reported tumor patterns, perceived diagnostic availability, and self-reported preparedness. Mammary and cutaneous/subcutaneous tumors were prominent in both sources, but the ordering of the remaining categories differed. These findings describe submitted tumors and practitioner-reported experience and should not be interpreted as estimates of population-level tumor occurrence; likewise, the high proportion of malignant diagnoses applies only to the submitted material. The unfavorable diagnostic-availability ratings and geographically concentrated submission catchment suggest potential gaps between clinical recognition, histopathological confirmation, and systematic recording. Standardized submission and coding procedures, more accessible specimen-transport and formal pathology-referral pathways, broader geographic participation, continuing professional education, and the progressive development of a companion-animal cancer-registration system would strengthen tumor documentation and surveillance in Algeria. 

## Supplementary Information

Additional file 1: Supplementary tables. This workbook contains supplementary tables. Supplementary Table S1 provides the complete ungrouped histopathological diagnosis list of the 86 tumor submissions, including counts and percentages. Supplementary Table S2 provides coded multiple-response practitioner-reported tumor-category reports from the companion-animal questionnaire subset, including counts and percentages. Supplementary Table S3 shows the full geographic distribution of questionnaire responses and histopathology-confirmed tumor submissions. It also reports the composition of the “Other wilayas” group displayed in Figure 2.

Additional file 2: Questionnaire instrument used for the veterinarian survey. Note. The questionnaire was administered to veterinarians in French. An English translation is provided first for reviewer and reader accessibility, followed by the original French version. Part A. English translation of the questionnaire. Part B. Original French questionnaire administered to respondents.

## Data Availability

All summary data supporting the findings are included in the article and its additional files. The underlying questionnaire and clinical-histopathological datasets are not publicly available because they contain non-public clinical, diagnostic-submission, and practice-related information. De-identified data required to reproduce the aggregated analyses may be made available by the corresponding author upon reasonable request and subject to confidentiality considerations.

## Abbreviations

CTVT: Canine transmissible venereal tumor
HE: Hematoxylin and eosin
IQR: Interquartile range
