# Supplementary material for: Spontaneous tumors in dogs and cats in Algeria: a dual-source descriptive study of practitioner-reported patterns and histopathology-submitted cases
Source: BMC Vet Res. 2026 Aug 24;22:612. doi: 10.1186/s12917-026-05856-3 (PMC13675969; doi:10.1186/s12917-026-05856-3)
Supplement: Supplementary file 1 [file 12917_2026_5856_MOESM2_ESM.pdf]

Part A: Questionnaire on Tumors in Domestic Carnivores in Algeria: Practices and Perceptions of Veterinarians

Purpose of the survey

This survey aims to collect information on the knowledge, diagnostic and therapeutic practices, and perceptions of veterinarians in Algeria regarding tumors in domestic carnivores, specifically dogs and cats.

The objective is to better understand the management of these conditions in daily practice and the challenges encountered by veterinarians.

We respect your confidentiality; your profile/email address will remain anonymous.

1. Veterinarian profile

1.1. What is your age?

- Under 30 years
- 30–40 years
- 41–50 years
- 51–60 years
- Over 60 years

1.2. In which region of Algeria do you mainly practice?

- Algiers
- Oran
- Constantine
- Annaba
- Setif
- Other (please specify): _____________

1.3. What type of practice facility do you work in?

- Urban veterinary clinic
- Rural veterinary practice
- Other (please specify): _____________

1.4. How many veterinarians work in your practice/facility?

- 1 veterinarian
- 2–3 veterinarians
- 4–5 veterinarians
- More than 5 veterinarians

1.5. What type of animals do you mainly treat?

- Dogs and cats
- Production animals (ruminants)
- Equids
- Other animals (please specify): _____________

1.6. How long have you been practicing veterinary medicine?

…………………………………..

1.7. Do you have specific experience in treating tumors in domestic carnivores?

- Yes, I regularly treat tumor cases
- Yes, but only in specific cases
- No, this is not a specialty that I manage often

2. Frequency and types of tumors observed

2.1. On a scale from 1 to 5, how often do you diagnose tumors in domestic carnivores in your daily practice?

(1 = Very rarely, 5 = Very frequently)

- 1 - Very rarely
- 2 - Rarely
- 3 - Sometimes
- 4 - Frequently
- 5 - Very frequently

2.2. Which types of tumors do you diagnose most frequently in dogs and cats?

(Multiple answers possible)

- Cutaneous tumors
- Mammary tumors
- Lymphomas (lymph nodes)
- Bone tumors
- Pulmonary tumors
- Digestive tumors
- Other (please specify): _____________

2.3. In your opinion, how frequent are tumors in dogs compared with cats in your practice?

- More frequent in dogs
- More frequent in cats
- Approximately equal in both species

2.4. Which types of tumors do you diagnose more frequently in dogs?

(Multiple answers possible)

- Cutaneous tumors
- Mammary tumors
- Bone tumors
- Lymphomas
- Pulmonary tumors
- Digestive tumors
- Other (please specify): _____________

2.5. Which types of tumors do you diagnose more frequently in cats?

(Multiple answers possible)

- Cutaneous tumors
- Mammary tumors
- Bone tumors
- Lymphomas
- Pulmonary tumors
- Digestive tumors
- Other (please specify): _____________

2.6. For the same tumor type, how do you assess its severity in dogs compared with cats?

- Tumors in dogs are generally more severe
- Tumors in cats are generally more severe
- Severity is similar in both species

3. Diagnosis and treatment of tumors

3.1. What are the main diagnostic methods you use to identify tumors in domestic carnivores?

(Multiple answers possible)

- Clinical examination
- Mass biopsy (histopathology)
- Cytology (smears, aspirates, etc.)
- Imaging
- Blood tests (tumor markers, etc.)
- Other (please specify): _____________

3.2. What is your treatment of choice for mammary tumors in dogs and cats?

- Surgery
- Chemotherapy
- Radiotherapy
- Palliative treatments
- Other (please specify): _____________

3.3. For tumor treatment, what difference do you observe between dogs and cats regarding response to chemotherapy?

- Dogs respond better to chemotherapy
- Cats respond better to chemotherapy
- The response is similar in both species
- I do not use chemotherapy in my practice

3.4. Are dog owners more inclined to pursue costly or complex treatments for tumors than cat owners?

- Yes, dog owners are generally more inclined
- No, cat owners are generally more inclined
- Both are equally inclined to pursue complex treatments

4. Perceptions and continuing education

4.1. How do you rate the availability of diagnostic resources (equipment, training, etc.) for tumor management in Algeria?

- Very satisfactory
- Satisfactory
- Insufficient
- Not at all satisfactory

4.2. To what extent do you feel prepared to diagnose and treat tumors in domestic carnivores?

- Very well prepared
- Well prepared
- Fairly prepared
- Moderately prepared
- Not well prepared

4.3. Are you interested in specialized training sessions or conferences on tumor management in carnivores?

- Yes
- No
- Maybe (please specify): _____________

5. Conclusion

5.1. In your opinion, what are the main challenges in managing tumors in domestic carnivores in Algeria?

- Lack of advanced diagnostic resources
- Lack of specialists in veterinary oncology
- High cost of treatments
- Lack of awareness among animal owners
- Other (please specify): _____________

5.2. Would you like to add any comments or suggestions regarding the management of tumors in domestic carnivores in Algeria?

Acknowledgements

Thank you for taking the time to participate in this survey. Your responses are valuable and will contribute to improving the management of tumors in domestic carnivores in Algeria.

Part B : Enquête sur les tumeurs chez les carnivores domestiques en Algérie : pratiques et perceptions des vétérinaires

Objectif de l'enquête

Cette enquête vise à recueillir des informations sur les connaissances, les pratiques diagnostiques et thérapeutiques, ainsi que les perceptions des vétérinaires en Algérie concernant les tumeurs chez les carnivores domestiques (chiens et chats).

L'objectif est de mieux comprendre la gestion de ces pathologies dans la pratique quotidienne et les défis rencontrés par les vétérinaires.

Nous respectons votre confidentialité, votre profil/adresse mail est anonyme.

1. Profil du vétérinaire

1.1. Quel est votre âge ?

- Moins de 30 ans
- 30-40 ans
- 41-50 ans
- 51-60 ans
- Plus de 60 ans

1.2. Dans quelle région d'Algérie exercez-vous principalement ?

- Alger
- Oran
- Constantine
- Annaba
- Sétif
- Autre (préciser) : _____________

1.3. Quel est le type de structure dans laquelle vous exercez ?

- Clinique vétérinaire de ville
- Cabinet vétérinaire rural
- Autre (préciser) : _____________

1.4. Combien de vétérinaires travaillent dans votre structure ?

- 1 vétérinaire
- 2-3 vétérinaires
- 4-5 vétérinaires
- Plus de 5 vétérinaires

1.5. Quel type d'animaux traitez-vous principalement ?

- Chiens et chats
- Animaux de rente (ruminants)
- Équidés
- Autres animaux (précisez) : _____________

1.6. Depuis quand exercez-vous la médecine vétérinaire ?

…………………………………..

1.7. Avez-vous une expérience particulière dans le traitement des tumeurs chez les carnivores ?

- Oui, je traite régulièrement des cas de tumeurs
- Oui, mais seulement dans des cas spécifiques

- Non, ce n’est pas une spécialité que je gère souvent

2. Fréquence et types de tumeurs observées

2.1. Sur une échelle de 1 à 5, à quelle fréquence diagnostiquez-vous des tumeurs chez les carnivores domestiques dans votre pratique quotidienne ?

(1 = Très rarement, 5 = Très fréquemment)

- 1 - Très rarement
- 2 - Rarement
- 3 - Parfois
- 4 - Fréquemment
- 5 - Très fréquemment

2.2. Quels types de tumeurs diagnostiquez-vous le plus fréquemment chez les chiens et les chats ?

(Plusieurs réponses possibles)

- Tumeurs cutanées
- Tumeurs mammaires
- Lymphomes (ganglions)
- Tumeurs osseuses
- Tumeurs pulmonaires
- Tumeurs digestives
- Autres (précisez) : _____________

2.3. Selon vous, quelle est la fréquence des tumeurs chez les chiens par rapport aux chats dans votre pratique ?

- Plus fréquentes chez les chiens
- Plus fréquentes chez les chats
- À peu près égales chez les deux

2.4. Quels types de tumeurs diagnostiquez-vous plus fréquemment chez les chiens ?

(Plusieurs réponses possibles)

- Tumeurs cutanées
- Tumeurs mammaires
- Tumeurs osseuses
- Lymphomes
- Tumeurs pulmonaires
- Tumeurs digestives
- Autres (précisez) : _____________

2.5. Quels types de tumeurs diagnostiquez-vous plus fréquemment chez les chats ?

(Plusieurs réponses possibles)

- Tumeurs cutanées
- Tumeurs mammaires
- Tumeurs osseuses
- Lymphomes
- Tumeurs pulmonaires
- Tumeurs digestives
- Autres (précisez) : _____________

2.6. Pour une même tumeur, comment évaluez-vous sa gravité chez les chiens par rapport aux chats ?

- Les tumeurs chez les chiens sont généralement plus graves
- Les tumeurs chez les chats sont généralement plus graves

- La gravité est similaire pour les deux espèces

3. Diagnostic et traitement des tumeurs

3.1. Quels sont les principaux moyens diagnostiques que vous utilisez pour identifier les tumeurs chez les carnivores domestiques ?

(Plusieurs réponses possibles)

- Examen clinique
- Biopsie de la masse (anatomopathologie)
- Cytologie (frottis, aspirats, etc.)
- Imagerie
- Tests sanguins (marqueurs tumoraux, etc.)
- Autres (préciser) : _____________

3.2. Quel est votre traitement de choix pour les tumeurs mammaires chez les chiens et les chats ?

- Chirurgie
- Chimiothérapie
- Radiothérapie
- Traitements palliatifs
- Autres (précisez) : _____________

3.3. Pour le traitement des tumeurs, quelle différence observez-vous entre les chiens et les chats concernant la réponse à la chimiothérapie ?

- Les chiens réagissent mieux à la chimiothérapie
- Les chats réagissent mieux à la chimiothérapie
- La réponse est similaire pour les deux espèces
- Je n'utilise pas la chimiothérapie dans ma pratique

3.4. Les propriétaires de chiens sont-ils plus enclins à poursuivre des traitements coûteux ou complexes pour les tumeurs que ceux des chats ?

- Oui, les propriétaires de chiens sont généralement plus enclins
- Non, les propriétaires de chats sont généralement plus enclins
- Les deux sont également enclins à poursuivre des traitements complexes

4. Perceptions et formation continue

4.1. Comment évaluez-vous la disponibilité des ressources diagnostiques (équipements, formations, etc.) pour le traitement des tumeurs en Algérie ?

- Très satisfaisante
- Satisfaisante
- Insuffisante
- Pas du tout satisfaisante

4.2. Dans quelle mesure vous sentez-vous préparé à diagnostiquer et traiter les tumeurs chez les carnivores domestiques ?

- Très bien préparé
- Bien préparé
- Assez préparé
- Moyennement préparé
- Pas bien préparé

4.3. Êtes-vous intéressé par des formations ou des conférences spécialisées sur la gestion des tumeurs chez les carnivores ?

- Oui
- Non
- Peut-être (précisez) : _____________

5. Conclusion

5.1. Quels sont, selon vous, les principaux défis dans la gestion des tumeurs chez les carnivores en Algérie ?

- Manque de ressources diagnostiques avancées
- Manque de spécialistes en oncologie vétérinaire
- Coût élevé des traitements
- Manque de sensibilisation des propriétaires d'animaux
- Autres (précisez) : _____________

5.2. Souhaitez-vous ajouter des commentaires ou des suggestions concernant la prise en charge des tumeurs chez les carnivores domestiques en Algérie ?

Remerciements

Merci d'avoir pris le temps de participer à cette enquête. Vos réponses sont précieuses et contribueront à améliorer la prise en charge des tumeurs chez les carnivores domestiques en Algérie.
